# GeneGPT: Augmenting Large Language Models with Domain Tools for Improved Access to Biomedical Information

**Published:** 2023-05-16

**Authors:** Qiao Jin, Yifan Yang, Qingyu Chen, Zhiyong Lu

**Affiliations:** ♣ National Library of Medicine, National Institutes of Health; ♡ University of Maryland, College Park

## Abstract

While large language models (LLMs) have been successfully applied to various tasks, they still face challenges with hallucinations. Augmenting LLMs with domain-specific tools such as database utilities can facilitate easier and more precise access to specialized knowledge. In this paper, we present GeneGPT, a novel method for teaching LLMs to use the Web APIs of the National Center for Biotechnology Information (NCBI) for answering genomics questions. Specifically, we prompt Codex to solve the GeneTuring tests with NCBI Web APIs by in-context learning and an augmented decoding algorithm that can detect and execute API calls. Experimental results show that GeneGPT achieves state-of-the-art performance on eight tasks in the GeneTuring benchmark with an average score of 0.83, largely surpassing retrieval-augmented LLMs such as the new Bing (0.44), biomedical LLMs such as BioMedLM (0.08) and BioGPT (0.04), as well as GPT-3 (0.16) and ChatGPT (0.12). Our further analyses suggest that: (1) API demonstrations have good cross-task generalizability and are more useful than documentations for in-context learning; (2) GeneGPT can generalize to longer chains of API calls and answer multi-hop questions in GeneHop, a novel dataset introduced in this work; (3) Different types of errors are enriched in different tasks, providing valuable insights for future improvements.

## 1 Introduction

Large language models (LLMs) such as PaLM (Chowdhery et al., 2022) and GPT-4 (OpenAI, 2023) have shown great success on a wide range of general-domain Natural Language Processing (NLP) tasks. They also achieve state-of-the-art (SOTA) performance on domain-specific tasks like biomedical question answering (Singhal et al., 2022; Liévin et al., 2022; Nori et al., 2023). However, since there is no intrinsic mechanism for autoregressive LLMs to “consult” with any source of truth, they can generate plausible-sounding but incorrect content (Ji et al., 2023). To tackle the hallucination issue, various studies have been proposed to augment LLMs (Mialon et al., 2023) by either conditioning them on retrieved relevant content (Guu et al., 2020; Lewis et al., 2020; Borgeaud et al., 2022) or allowing them to use other external tools such as program APIs (Gao et al., 2022; Parisi et al., 2022; Schick et al., 2023; Qin et al., 2023).

In this work, we propose to teach LLMs to use the Web APIs of the National Center for Biotechnology Information (NCBI). NCBI provides API access to its entire biomedical databases and tools, including Entrez Programming Utilities (E-utils) and Basic Local Alignment Search Tool (BLAST) URL API (Altschul et al., 1990;Schuler et al., 1996;Sayers et al., 2019). Enabling LLMs to use NCBI Web APIs can provide easier and more precise access to biomedical information, especially for users who are inexperienced with the database systems. More importantly, Web APIs can relieve users from locally implementing functionalities, maintaining large databases, and heavy computation burdens because the only requirement for using Web APIs is an internet connection.

We introduce GeneGPT, a novel method that prompts Codex (Chen et al., 2021) to use NCBI Web APIs by in-context learning (Brown et al., 2020). GeneGPT consists of two main modules: (a) a specifically designed prompt that consists of documentations and demonstrations of API usage, and (b) an inference algorithm that integrates API calls in the Codex decoding process. We evaluate GeneGPT on GeneTuring (Hou and Ji, 2023), a question answering (QA) benchmark for genomics, and compare GeneGPT to a variety of other LLMs such as the new Bing^1^, ChatGPT^2^, and BioGPT (Luo et al., 2022). GeneGPT achieves the best performance on eight GeneTuring tasks with an average score of 0.83, which is remarkably higher than the previous SOTA (0.44 by New Bing). In addition, we systematically characterize GeneGPT and find that: (1) API demonstrations are more useful than documentations for in-context learning; (2) GeneGPT generalizes to longer chains of subquestion decomposition and API calls with simple demonstrations; (3) GeneGPT makes specific errors that are enriched for each task.

In summary, our contributions are three-fold:

1. We introduce GeneGPT, a novel method that uses NCBI Web APIs to answer biomedical questions. To the best of our knowledge, this is the first study on augmenting LLMs with domain-specific Web API tools.
2. GeneGPT achieves SOTA performance on 8 tasks in the GeneTuring benchmark, largely outperforming previous best results by 88% (0.83 v.s. 0.44 set by the new Bing).
3. We conduct experiments to further characterize GeneGPT, including ablation, probing, and error analyses. We also contribute a novel GeneHop dataset, and use it to show that GeneGPT can perform chain-of-thought API calls to answer multi-hop genomics questions.

## 2 GeneGPT

In this section, we first introduce the general functions and syntax of NCBI Web APIs (§2.1). We then describe two key components of GeneGPT: its prompt design for in-context learning (§2.2) and the inference algorithm (§2.3).

### 2.1 NCBI Web APIs

We utilize NCBI Web APIs of E-utils^3^ that provide access to biomedical databases and the BLAST tool^4^ for DNA sequence alignment. Web API calls are implemented by the urllib library in Python.

### E-utils.

It is the API for accessing the Entrez portal (Schuler et al., 1996), which is a system that covers 38 NCBI databases of biomedical data such as genes and proteins (Sayers et al., 2019). The E-utils API provides a fixed URL syntax for rapidly retrieving such biomedical information. Specifically, the base URL for an E-utils request is “https://eutils.ncbi.nlm.nih.gov/entrez/eutils/{function}.fcgi”, where function can be esearch, efetch, or esummary. Typically, the user first calls esearch to get the unique database identifiers of a given query term. Then, efetch or esummary can be called to get the full records or text summaries of a given list of identifiers returned by esearch. Important arguments in the URL request include the search term or ids (term or id), the database to use (db), the maximum number of returned items (retmax), and the return format (retmode).

### BLAST URL API.

BLAST takes as input a sequence of nucleotides or amino acids and finds the most similar sequences in the database (Altschul et al., 1990; Boratyn et al., 2013). The results can be used to infer relationships between sequences or identify members of gene families. The BLAST API allows users to submit queries to find regions of similarities between nucleotide or protein sequences to existing databases using the BLAST algorithm on NCBI servers. The base URL for the BLAST URL API is “https://blast.ncbi.nlm.nih.gov/blast/Blast.cgi”. By sending different parameters to this API, the user can submit and retrieve queries that are executed by NCBI web servers. Every call to the API must include a CMD parameter that defines the type of the call. When submitting queries using CMD=Put, the user can specify the querying database with the DATABASE parameter, the searching program with the PROGRAM parameter, and the query sequence with the QUERY parameter. The user will get an RID after the CMD=Put API call, and can make another API call with the Get command and the returned RID to retrieve its BLAST results.

### 2.2 In-context learning

**Figure 1:**
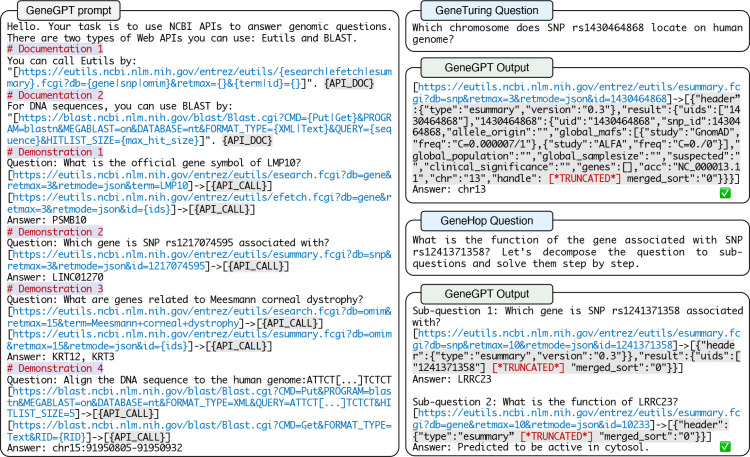
**Left:** GeneGPT uses NCBI Web API documentations and demonstrations in the prompt for in-context learning. **Right:** Examples of GeneGPT answering GeneTuring and GeneHop questions with NCBI Web APIs.

We teach an LLM to use NCBI Web APIs through in-context learning with an engineered prompt. Figure 1 shows an example of the GeneGPT prompt, which is composed of four modules: 1. an instruction; 2. API documentations; 3. API demonstrations; 4. a test question. The first three parts are fixed for all tasks, while the last one is task-specific.

1. **Instruction:** The prompt starts with an overall task description (“Your task is to use NCBI APIs to answer genomic questions.”). It is then followed by documentations and demonstrations of API usage summarized in Table 1.
2. **Documentations (Dc.)** provide natural language descriptions of the API functionality, general syntax, and argument choices. We include one for the E-utils API (Dc.1) and one for the BLAST tool (Dc.2).
3. **Demonstrations (Dm.)** are concrete examples of using NCBI Web APIs to solve questions. Based on questions in the GeneTuring tasks, we manually write four demonstrations that cover four functions (esearch, efetch, esummary, blastn) and four databases (gene, snp, omim, nt) of E-utils and BLAST. The API URLs and the call results are marked up by “[ ]”, with a special “–>” symbol inserted in between that serves as an indicator for API calls.
4. **Test question:** The specific test question is then appended to the end of the prompt.

While the initial GeneGPT uses all documentations and demonstrations (denoted as GeneGPT-full in Table 2), we find through analyses in §4.1 that GeneGPT can work well with only two demonstrations (denoted as GeneGPT-slim) on all tasks.

### 2.3 Inference algorithm

The GeneGPT inference algorithm is briefly shown in Algorithm 1. Specifically, we first append the given question to the prompt (described in §2.2) and feed the concatenated text to Codex (code-davinci-002,Chen et al. (2021)) with a temperature of 0. We choose to use Codex for two reasons: (1) it is pre-trained with code data and shows better code understanding abilities, which is crucial in generating the URLs and interpreting the raw API results; (2) its API has the longest (8k tokens) context length among all available models so that we can fit the demonstrations in.

**Algorithm 1:** GeneGPT inference algorithm

**Input:** question
**Model:** Codex (code-davinci-002)
**Output:** answer
prompt ← header + demonstrations + question
finished ← False
**while** not finished **do**
next token ← Codex(prompt)
prompt ← prompt + next token
**if** next token is “ – > ” **then**
url ← extractLastURL(prompt)
result ← callWebAPI(url)
prompt ← prompt + result
**else if** next token is “\n\n” **then**
answer ← extractAnswer(prompt)
finished ← True
**end if**
**end while**

We discontinue the text generation process when the special “–>” symbol is detected, which is the indication for an API call request. Then we extract the last URL and call the NCBI Web API with it. The raw execution results will be appended to the generated text, and it will be fed to Codex to continue the generation. When “\n\n”, an answer indicator used in the demonstrations, is generated, we will stop the inference and extract the answer after the generated “Answer: ”.

## 3 Experiments

### 3.1 GeneTuring

The GeneTuring benchmark (Hou and Ji, 2023) contains 12 tasks, and each task has 50 question-answer pairs. We use 9 GeneTuring tasks that are related to NCBI resources to evaluate the proposed GeneGPT model, and the QA samples are shown in Appendix B. The chosen tasks are classified into four modules and briefly described in this section.

#### Nomenclature:

This is about gene names. We use the gene alias task and the gene name conversion task, where the objective is to find the official gene symbols for their non-official synonyms.

#### Genomics location:

The tasks are about the locations of genes, single-nucleotide polymorphism (SNP), and their relations. We include the gene location, SNP location, and gene SNP association tasks. The first two tasks ask for the chromosome locations (e.g., “chr2”) of a gene or an SNP, and the last one asks for related genes for a given SNP.

#### Functional analysis:

It asks for gene functions. We use the gene disease association task where the goal is to return related genes for a given disease, and the protein-coding genes task which asks whether a gene is a protein-coding gene or not.

#### Sequence alignment:

The tasks query specific DNA sequences. We use the DNA sequence alignment to human genome task and the DNA sequence alignment to multiple species task. The former maps an DNA sequence to a specific human chromosome, while the latter maps an DNA sequence to a specific species (e.g. “zebrafish”).

### 3.2 Compared methods

We evaluate two settings of GeneGPT, a full setting (GeneGPT-full) where all prompt components are used, as well as a slim setting (GeneGPT-slim) inspired by our ablation and probing analyses (§4.1) where only Dm.1 and Dm.4 are used.

We compare GeneGPT with various baselines evaluated by Hou and Ji (2023), including general-domain GPT-based (Radford et al., 2018) LLMs such as GPT-2 (Radford et al., 2019), GPT-3 (text-davinci-003) (Brown et al., 2020), and ChatGPT^5^, GPT-2-sized biomedical domain-specific LLMs such as BioGPT (Luo et al., 2022) and BioMedLM^6^, as well as the new Bing^7^, a retrieval-augmented LLM that has access to relevant web pages retrieved by Bing.

### 3.3 Evaluation

For the performance of the compared methods, we directly use the results reported in the original benchmark that are manually evaluated.

To evaluate our proposed GeneGPT method, we follow the general criteria but perform automatic evaluations. Specifically, we only consider *exact* matches between model predictions and the ground truth as correct predictions for all nomenclature and genomics location tasks. For the gene disease association task, we measure the recall as in the original dataset but based on *exact* individual gene matches. For the protein-coding genes task and the DNA sequence alignment to multiple species task, we also consider *exact* matches as correct after applying a simple vocabulary mapping that converts model-predicted “yes”/“no” to “TRUE”/“NA” and Latin species names to their informal names (e.g., “*Saccharomyces cerevisiae*” to “yeast”), respectively. For the DNA sequence alignment to human genome task, we give correct chromosome mapping but incorrect position mapping a score of 0.5 (e.g., chr8:7081648–7081782 v.s. chr8:1207812–1207946), since the original task does not specify a reference genome. Overall, our evaluation of GeneGPT is more strict than the original evaluation of other LLMs in Hou and Ji (2023), which performs manual evaluation and might consider non-exact matches as correct.

### 3.4 Main results

Table 2 shows the performance of GeneGPT on the GeneTuring tasks in comparison with other LLMs. For GeneGPT, tasks with “*” in Table 2 are one-shot where one instance is used as API demonstration, and the other tasks are zero-shot. For the compared LLMs, all tasks are zero-shot.

#### Nomenclature:

GeneGPT achieves state-of-the-art (SOTA) performance on both the one-shot gene alias task with an accuracy of 0.84 and the zero-shot gene name conversion task with an accuracy of 1.00. On average, GeneGPT outperforms New Bing by a large margin (0.92 v.s. 0.76). All other GPT models have accuracy scores of less than 0.10 on the nomenclature tasks.

#### Genomic location:

GeneGPT also achieves SOTA performance on all genomic location tasks, including the gene SNP association task (1.00) gene location task (0.66) and the SNP location task (1.00). While the New Bing is comparable to GeneGPT on gene location (0.61 v.s. 0.66), its performance on the two SNP-related tasks is close to 0. Again, most other LLMs score less than 0.10. Notably, while all genomics location tasks are zero-shot for GeneGPT-slim, it performs comparably to GeneGPT-full which uses one gene SNP association demonstration. This indicates that API demonstrations have strong cross-task generalizability.

#### Functional analysis:

The new Bing performs better functional analysis tasks than the proposed GeneGPT (average score: 0.91 v.s. 0.84), which is probably because many web pages related to gene functions can be retrieved by the Bing search engine. We also note that other LLMs, especially GPT-3 and ChatGPT, perform moderately well and much better than they perform on other tasks. This might also be due to the fact that many gene-function-related texts are included in their pre-training corpora.

#### Sequence alignment:

GeneGPT performs much better with an average score of 0.66 than all other models including the new Bing (0.00), which essentially fails on the sequence alignment tasks. This is not very surprising since sequence alignment is easy with the BLAST tool, but almost impossible for an auto-regressive LLM even with retrieval augmentation as the input sequences are too specific to be indexed by a search engine.

Although evaluated under a more strict setting (§3.3), GeneGPT achieves a macro-average performance of 0.83 which is much higher than other compared LLMs including New Bing (0.44). Overall, GeneGPT achieves new SOTA performance on all 2 one-shot tasks and 6 out of 7 zero-shot tasks and is outperformed by New Bing only on the gene disease association task.

## 4 Discussions

We have shown that GeneGPT largely surpasses various LLMs on the GeneTuring benchmark. In this section, we further characterize GeneGPT by studying three research questions (RQ):

**RQ1**: What is the importance of each prompt component in GeneGPT?

**RQ2**: Can GeneGPT answer multi-hop questions by chain-of-thought API calls?

**RQ3**: What types of errors does GeneGPT make on each studied task?

### 4.1 RQ1: Component importance

We conduct ablation and probing experiments to study the importance of individual prompt components, including 2 documentations (Dc.1, Dc.2) and 4 demonstrations (Dm.1–4) described in §2.2.

**Figure 2:**
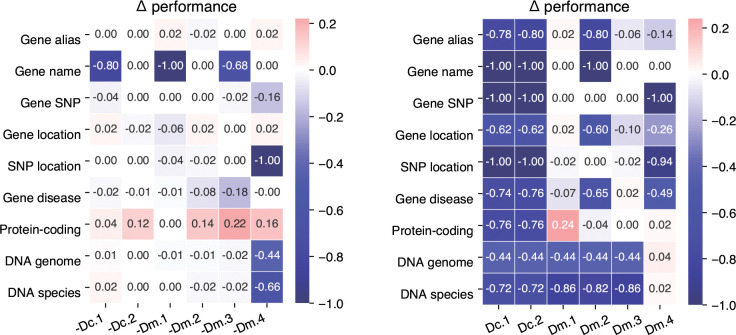
Performance changes of the ablation (**left**) and probing (**right**) experiments as compared to GeneGPT-full.

For ablation tests, we remove each component from GeneGPT-full and then evaluate the prompt. The results are shown in Figure 2 (left). Notably, the performance on the DNA to genome and species alignment tasks is only significantly decreased without the BLAST demonstration (Dm.4), but not affected by the ablation of the BLAST documentation (Dc.2). While the ablations of other components decrease the performance, most only affect one relevant task (e.g., Dm.1 and gene name conversion), which indicates a high level of redundancy of the prompt components.

For the probing experiments, we evaluate GeneGPT with only one prompt component to study the individual capability. The results are shown in Figure 2 (right). Overall, GeneGPT with only one documentation (Dc.1 or Dc.2) fails on all tasks. Surprisingly, with only one demonstration of the gene alias task (Dm.1) in the prompt, GeneGPT is able to perform comparably to GeneGPT-full on all tasks except the alignment ones. On the other hand, GeneGPT with only the BLAST demonstration (Dm.4) performs well on the two alignment tasks, which is somehow expected. These results suggest that GeneGPT with only two demonstrations (Dm.1 and Dm.4) in the prompt can generalize to all tasks in the GeneTuring benchmark. We denote this as GeneGPT-slim, and results in Table 2 show that with only two demonstrations, it outperforms the GeneGPT-full and achieves state-of-the-art overall results on GeneTuring.

### 4.2 RQ2: Multi-hop QA on GeneHop

Questions in the GeneTuring benchmark are single-hop and just require one step of reasoning, e.g., “Which gene is SNP rs983419152 associated with?”. However, many real-world biomedical questions are multi-hop that need more steps to answer (Jin et al., 2022). For example, to answer “What is the function of the gene associated with SNP rs983419152?”, the model should first get the associated gene name and then find its functions.

To test GeneGPT’s capability of answering multi-hop questions, we present **GeneHop**, a novel dataset that contains three new multi-hop QA tasks based on the GeneTuring benchmark: (a) **SNP gene function**, which asks for the function of the gene associated with a given SNP. (b) **Disease gene location**, where the task is to list the chromosome locations of the genes associated with a given disease. (c) **Sequence gene alias**, which asks for the aliases of the gene that contains a specific DNA sequence. Each task in GeneHop contains 50 questions, and the collection pipeline is detailed in Appendix C. For all tasks, we append the chain-of-thought instruction “Let’s decompose the question to sub-questions and solve them step by step.” after the test question (Wei et al., 2022b).

**Figure 3:**
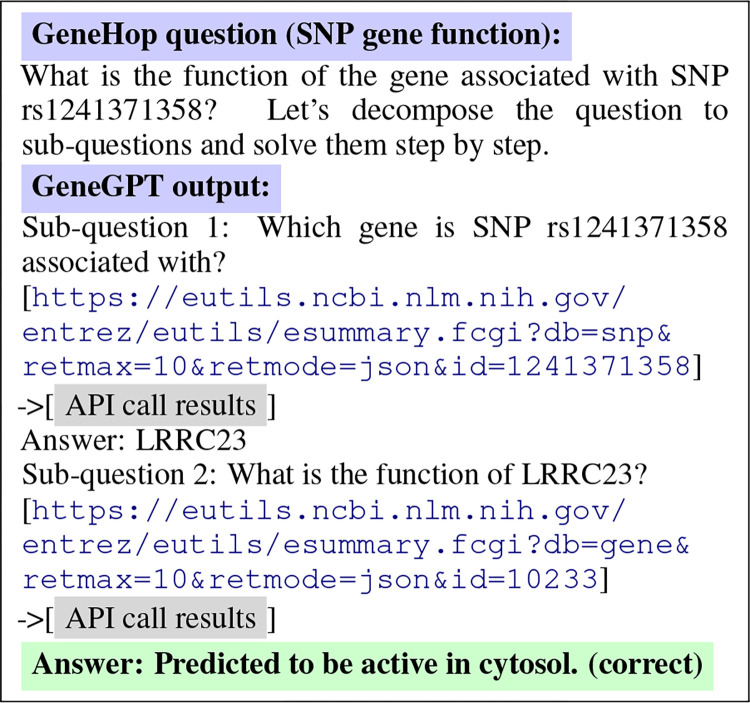
GeneGPT uses chain-of-thought API calls to answer a multi-hop question in GeneHop.

Figure 3 shows an example of GeneGPT to answer Task (a). In this case, GeneGPT successfully decomposes the multi-hop question into two sub-questions, and the sub-question 2 is based on the answer of the sub-question 1. Interestingly, GeneGPT uses a shortcut to answer sub-question 2: instead of first calling esearch and then calling esummary, GeneGPT finds the gene id in the API call results of sub-question 1 and directly calls esummary. This capability is not shown in the prompt but elicited by chain-of-thought API calls.

**Figure 4:**
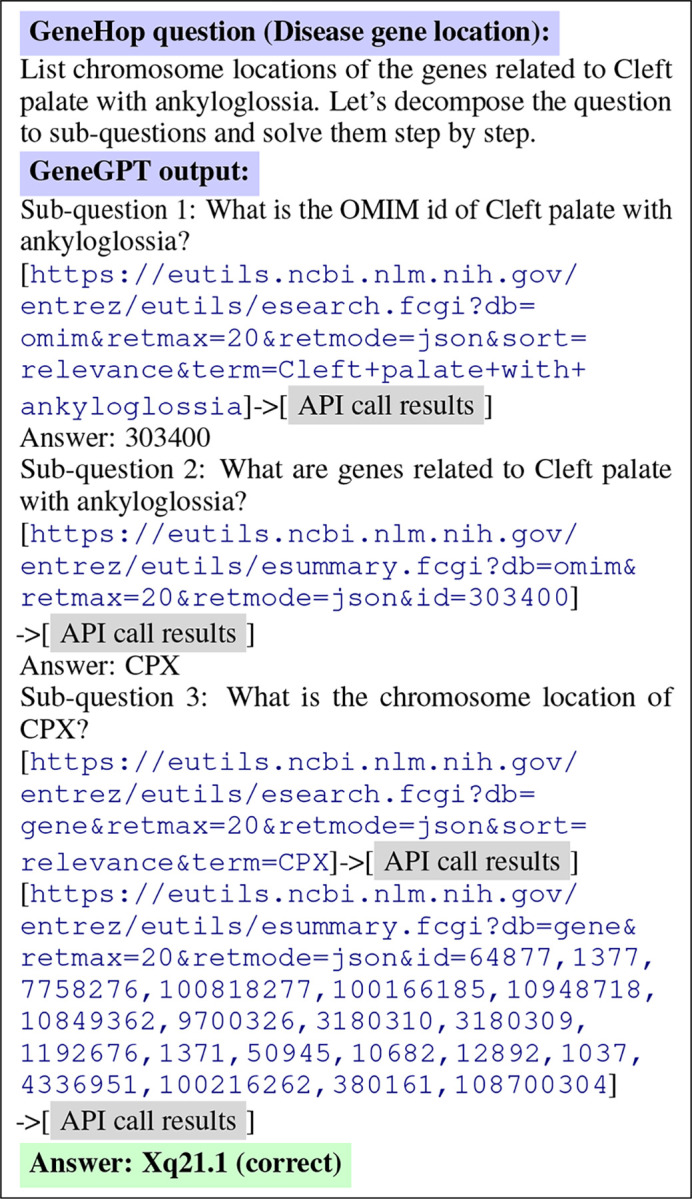
GeneGPT uses chain-of-thought API calls to answer a multi-hop question in GeneHop.

Figure 4 shows another example of GeneGPT answering Task (b), where GeneGPT successfully decomposes the multi-hop question and correctly calls the required APIs. Notably, the answering chain involves 3 sub-questions and 4 API calls, which are longer than all in-context demonstrations (1 single-hop question and 2 API calls at most). This ability to generalize to longer chains of thought is an important aspect of GeneGPT’s flexibility and usefulness for real-world applications.

We manually evaluate the results predicted by GeneGPT and compare it to the new Bing, which is the only baseline LLM that performs well on the single-hop GeneTuring benchmark due to its retrieval augmentation feature. The evaluation criteria are described in Appendix D. As shown in Table 3, while the new Bing outperforms GeneGPT on the disease gene location task, it is mostly using webpages that contain both the disease and location information without multi-hop reasoning. The new Bing fails to perform the other 2 tasks since the input information (SNP or sequence) is not indexed by Bing and can only be found in specialized databases. GeneGPT, on the other hand, performs moderately well on all 3 tasks, and achieves a much higher average score (0.50 v.s. 0.24).

### 4.3 RQ3: Error analysis

We manually study all errors made by GeneGPT and classify them into five types. Table 4 shows the count of each error type on the evaluate tasks: **E1:** using the wrong API or not using APIs, e.g., using the gene instead of the omin database for diseases; **E2:** using the right API but wrong arguments, e.g., passing terms to id; **E3:** not extracting the answer in the API result, most commonly seen in gene function extraction; **E4:** right API call but results do not contain the answer, where the question is not answerable with NCBI databases; and **O** includes other unclassified errors. Specific error examples are shown in Appendix E.

Our results suggest that different tasks have specific and enriched error types: simple tasks (alias and location) fail mostly because of **E4**; **E1** only happens in disease-related tasks; alignment tasks face more issues with BLAST interfaces and reference genomes (**O**); multi-hop tasks in GeneHop tend to have **E2** and **E3** in the reasoning chains.

## 5 Related work

### Large language models:

Recent studies have shown that scaling pre-trained LMs leads to performance improvement and potentially emergent abilities on various NLP tasks (Brown et al., 2020; Kaplan et al., 2020; Wei et al., 2022a; Chowdhery et al., 2022; OpenAI, 2023). However, such auto-regressive LLMs are still susceptible to hallucinations and generate erroneous content (Ji et al., 2023). Augmenting LLMs with external tools is a possible solution to this issue (Mialon et al., 2023).

### Tool augmentation:

Potential tools include: (1) search engines (Guu et al., 2020; Lewis et al., 2020; Borgeaud et al., 2022), also known as retrieval augmentation, exemplified by New Bing; (2) program APIs by in-context learning (Gao et al., 2022; Schick et al., 2023) or fine-tuning (Parisi et al., 2022; Schick et al., 2023). We present the first study on the in-context learning abilities of documentations and demonstrations of NCBI Web APIs.

### Biomedical question answering:

It is an essential step in clinical decision support (Ely et al., 2005) and biomedical knowledge acquisition (Jin et al., 2022). LLMs have been successfully applied to various biomedical QA tasks that are *knowledge*- or *reasoning*-intensive (Singhal et al., 2022; Liévin et al., 2022; Nori et al., 2023). However, autoregressive LLMs fail to perform *data*-intensive tasks which require the model to precisely store and recite database entries, such as the GeneTuring benchmark (Hou and Ji, 2023). Retrieval augmentation also falls short since specialized databases are usually not indexed by commercial search engines. GeneGPT solves this task by tool augmentation.

## 6 Conclusions

We present GeneGPT, a novel method that teaches LLMs to use NCBI Web APIs. It achieves SOTA performance on 8 GeneTuring tasks and can perform chain-of-thought API calls. Our results indicate that database utility tools might be superior to relevant web pages for augmenting LLMs to faithfully serve various biomedical information needs.

## Figures and Tables

**Table 1:** Summary of API usage documentations (Dc.1 and Dc.2) and demonstrations (Dm.1–4) in the GeneGPT prompt. Complete texts are shown in Appendix A.

Comp.	Documentation	Database	Function
Dc.1	E-utils	gene, snp, omim	esearch, efetch, esummary
Dc.2	BLAST	nt	blastn
Comp.	Demonstration	Database	Function
Dm.1	Alias	gene	esearch–> efetch
Dm.2	Gene SNP	snp	esummary
Dm.3	Gene disease	omim	esearch–> esummary
Dm.4	Alignment	nt	blastn

**Table 2:** Performance of GeneGPT compared to other LLMs on the GeneTuring benchmark.

GeneTuring task	GPT-2	BioGPT	BioMedLM	GPT-3	ChatGPT	New Bing	GeneGPT (ours)	
							-full	-slim

**Nomenclature**								
Gene alias	0.00	0.00	0.04	0.09	0.07	0.66	0.80 *	**0.84** *
Gene name conversion	0.00	0.00	0.00	0.00	0.00	0.85	**1.00**	**1.00**
Average	0.00	0.00	0.02	0.05	0.04	0.76	0.90	**0.92**

**Genomic location**								
Gene SNP association	0.00	0.00	0.00	0.00	0.00	0.00	**1.00** *	**1.00**
Gene location	0.01	0.04	0.12	0.09	0.09	0.61	0.62	**0.66**
SNP location	0.03	0.05	0.01	0.02	0.05	0.01	**1.00**	0.98
Average	0.01	0.03	0.04	0.04	0.05	0.21	0.87	**0.88**

**Functional analysis**								
Gene disease association	0.00	0.02	0.16	0.34	0.31	**0.84**	0.76 *	0.66
Protein-coding genes	0.00	0.18	0.37	0.70	0.54	0.97	0.76	**1.00**
Average	0.00	0.10	0.27	0.52	0.43	**0.91**	0.76	0.84

**Sequence alignment**								
DNA to human genome	0.02	0.07	0.03	0.00	0.00	0.00	**0.44** *	**0.44** *
DNA to multiple species	0.02	0.00	0.00	0.20	0.00	0.00	0.86	**0.88**
Average	0.02	0.04	0.02	0.10	0.00	0.00	0.65	**0.66**

**Overall average**	0.00	0.04	0.08	0.16	0.12	0.44	0.80	**0.83**

* One-shot learning for GeneGPT. **Bolded** and underlined numbers denote the highest and second-highest performance, respectively.

**Table 3:** Performance of multi-hop QA on GeneHop. We only compare GeneGPT with New Bing since other LLMs cannot even answer single-hop questions well.

GeneHop Task	New Bing	GeneGPT

SNP gene function	0.00	**0.55**
Disease gene location	**0.71**	0.67
Sequence gene alias	0.00	**0.28**

Average	0.24	**0.50**

**Table 4:** Counts of GeneGPT errors on different tasks.

GeneTuring Task	E1	E2	E3	E4	O

Gene alias	0	0	2	6	0
Gene location	0	0	0	17	0
SNP location	0	1	0	0	0
Gene disease association	15	0	0	3	2
DNA to human genome	0	0	7	0	42
DNA to multiple species	0	0	1	1	4

GeneHop Task	E1	E2	E3	E4	O

SNP gene function	0	0	29	0	0
Disease gene location	4	7	1	5	1
Sequence gene alias	0	30	8	0	0

**E1**: wrong API; **E2**: wrong arguments; **E3**: wrong comprehension; **E4**: unanswerable with API; **O**: others.

**Figure 5:**
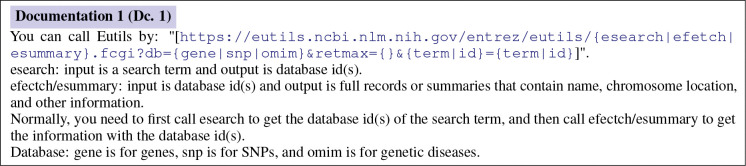
Documentation 1 (Dc.1) of the GeneGPT prompt. Dc.1 describes the functionality, general syntax, and argument choices of the NCBI E-utils API.

**Figure 6:**
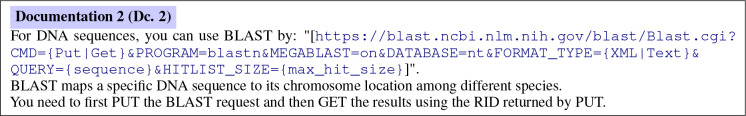
Documentation 2 (Dc.2) of the GeneGPT prompt. Dc.2 describes the functionality, general syntax, and argument choices of the BLAST API.

**Figure 7:**
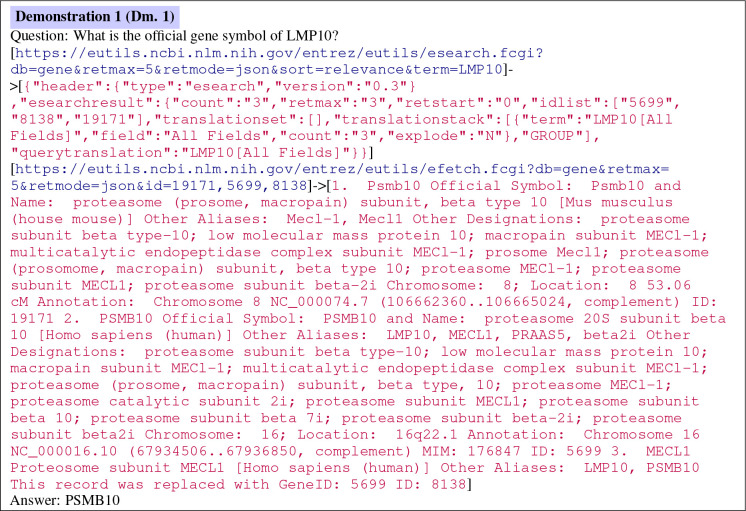
Demonstration 1 (Dm.1) of the GeneGPT prompt. The instance is chosen from the gene alias task in the GeneTuring benchmark. Links are actually called Web API URLs. Readers can directly click the link and get the API call result, which is inserted in the prompt.

**Figure 8:**
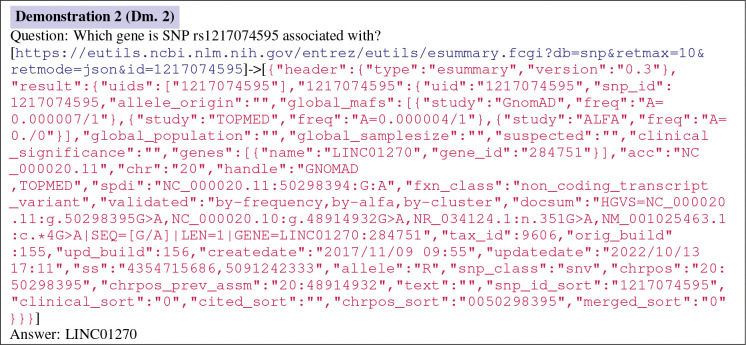
Demonstration 2 (Dm.2) of the GeneGPT prompt. The instance is chosen from the gene SNP association task in the GeneTuring benchmark. Links are actually called Web API URLs. Readers can directly click the link and get the API call result, which is inserted in the prompt.

**Figure 9:**
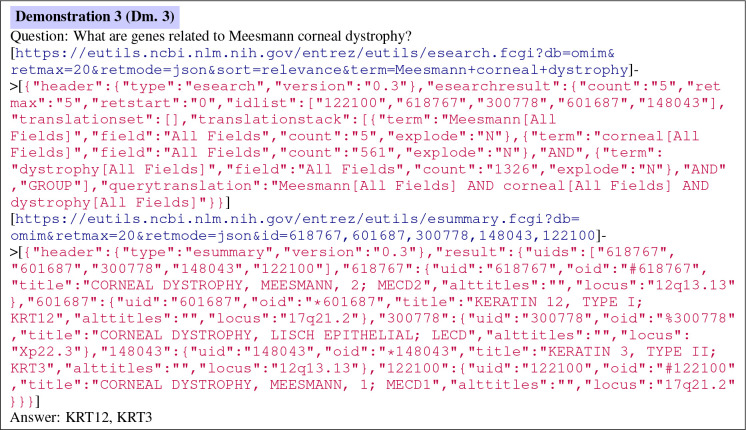
Demonstration 3 (Dm.3) of the GeneGPT prompt. The instance is chosen from the gene disease association task in the GeneTuring benchmark. Links are actually called Web API URLs. Readers can directly click the link and get the API call result, which is inserted in the prompt.

**Figure 10:**
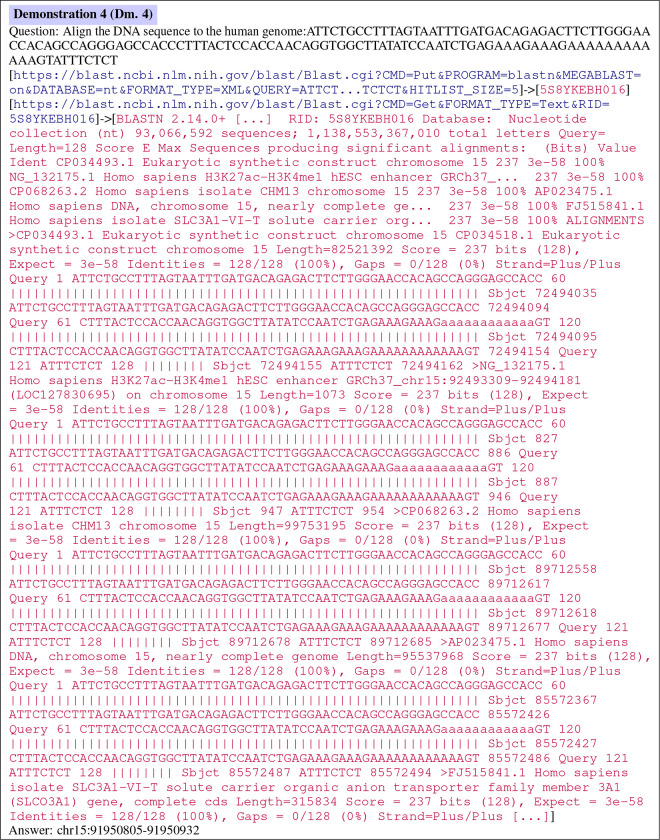
Demonstration 4 (Dm.4) of the GeneGPT prompt. The instance is chosen from the DNA to human genome alignment task in the GeneTuring benchmark. Links are actually called Web API URLs. Readers can directly click the link and get the API call result, which is inserted in the prompt. Since the BLAST Web API is about to be depricated and the first step returns an HTML page, we use a regular expression to extract the PID and append it to the GeneGPT input.

**Figure 11:**
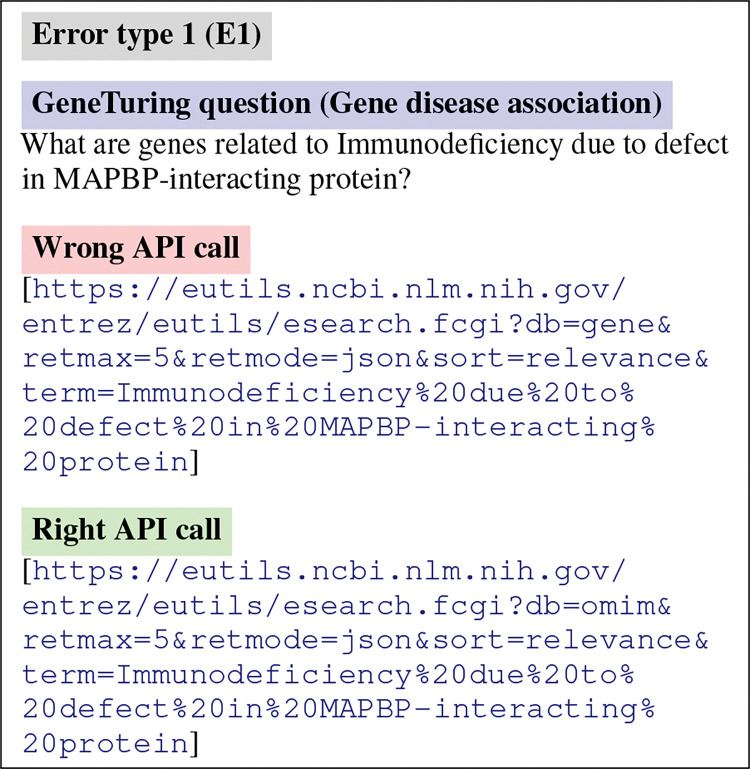
An example of Error type 1 (E1). The wrong API call uses gene database instead of the omin database used by the right API call.

**Figure 12:**
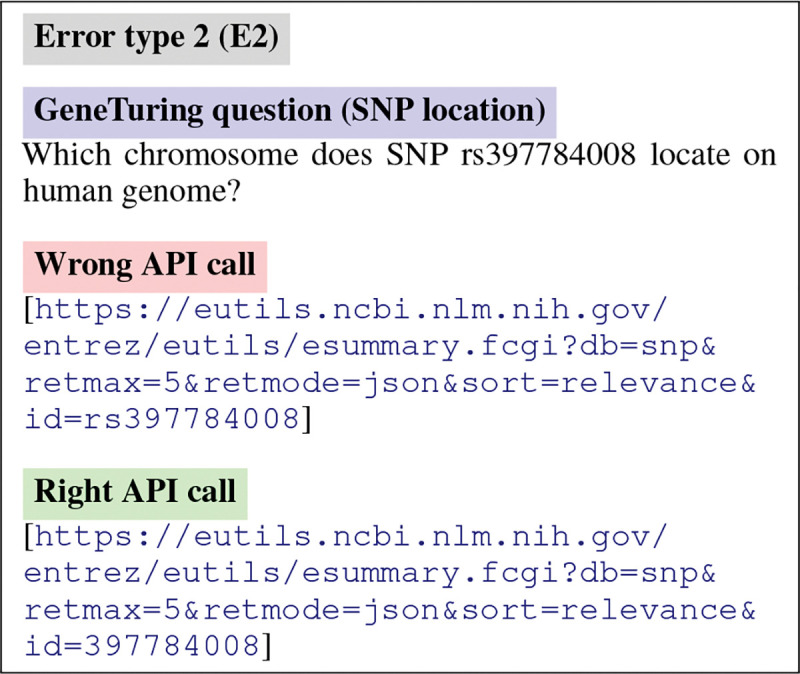
An example of Error type 2 (E2). The wrong API call passes all rs397784008 to the id argument, while the right API should only pass 397784008 without rs to the id argument.

**Figure 13:**
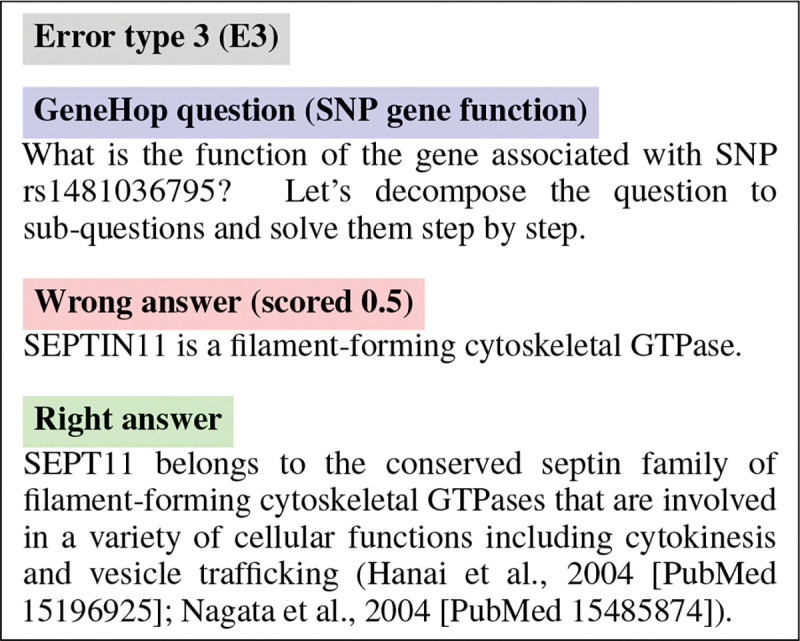
An example of Error type 3 (E3). The wrong answer only contains the protein family information without other details about its functions.

**Figure 14:**
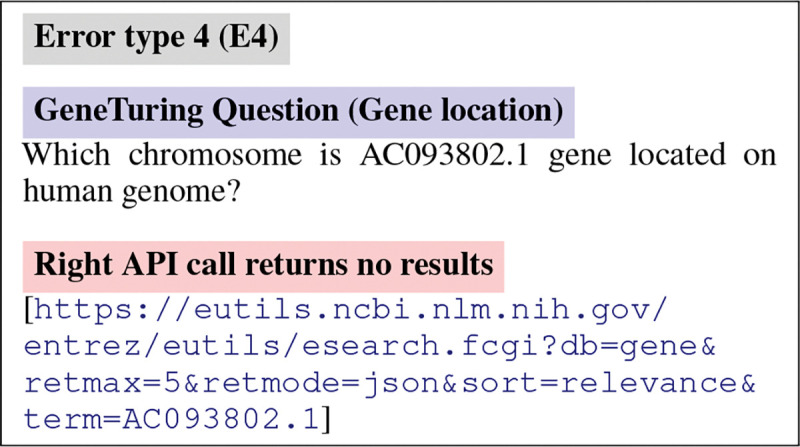
An example of Error type 4 (E4). The model makes the right API call but there is no entry returned from the API. The gene AC093802.1 is not indexed by the NCBI gene database.

## Footnotes

1
https://www.bing.com/new

2
https://chat.openai.com/

3
https://www.ncbi.nlm.nih.gov/books/NBK25501/

4
https://ncbi.github.io/blast-cloud/dev/api.html

5 https://chat.openai.com/ (Jan 31 version).

6 https://crfm.stanford.edu/2022/12/15/biomedlm.html, previously known as PubMedGPT.

7
https://www.bing.com/new

## A GeneGPT prompt

Here we show the exact texts of each prompt component described in Table 1, including two documentations Dc.1 (E-utils, Figure 5) and Dc.2 (BLAST, Figure 6) as well as four demonstrations Dm.1 (gene alias, Figure 7), Dm.2 (gene SNP association, Figure 8), Dm.3 (gene disease association, Figure 9), and Dm.4 (DNA to human genome alignment, Figure 10).

## B GeneTuring samples

Table 5 shows some sample question-answer pairs from the GeneTuring benchmark. The dataset is publicly available at https://www.biorxiv.org/content/10.1101/2023.03.11.532238v1.supplementary-material. We use the same questions sent to the new Bing as the test questions.

## C GeneHop collection

The GeneHop dataset contains three multi-hop tasks: SNP gene function, disease gene location, and sequence gene alias. We describe the collection of these tasks in this section. Table 6 shows several question-answer samples from the GeneHop dataset.

### SNP gene function:

The question template for this task is *“What is the function of the gene associated with SNP* {snp}*? Let’s decompose the question to sub-questions and solve them step by step.”*. We re-use the 50 {snp} from the gene SNP association task in the original GeneTuring benchmark. The ground-truth answer of the gene function is manually annotated: For each SNP, we first get its corresponding gene from the annotations of the gene SNP association task. We then check the gene information page^8^ and select its functional summary as the ground-truth answer.

### Disease gene location:

The question template for this task is *“List chromosome locations of the genes related to* {disease}. *Let’s decompose the question to sub-questions and solve them step by step.”*. Similarly, we re-use the 50 {disease} from the gene disease association task in the original GeneTuring benchmark. The ground-truth list of the chromosome locations is manually annotated: For each disease, we first get its corresponding genes from the annotations of the gene disease association task. We then check their NCBI gene information pages and label the cytogenetics locations (e.g., 21q22.3).

**Table 5:** Sample question-answer pairs of the GeneTuring tasks (Hou and Ji, 2023).

GeneTuring task	Question	Answer
Nomenclature		
Gene alias	What is the official gene symbol of SNAT6?	SLC38A6
Gene name conversion	Convert ENSG00000215251 to official gene symbol.	FASTKD5
Genomic location		
Gene SNP association	Which gene is SNP rs996319727 associated with?	USP39
Gene location	Which chromosome is FOXL2NB gene located on human genome?	chr3
SNP location	Which chromosome does SNP rs427884 locate on human genome?	chrll
Functional analysis		
Gene disease association	What are genes related to Bile acid malabsorption?	SLC10A2, SLC51B
Protein-coding genes	Is ATP5F1EP2 a protein-coding gene?	NA
Sequence alignment		
DNA to human genome	Align the DNA sequence to the human genome: AGGCCC TCACCT GGAAAT TACTTA CTCATG CTTCAT GACCCA GTTCAA ATTTTG TCACCT CTGTGA AACCTT CCCTGG GCCCCG TTGATC TCCTTG AAGGCA	chr7:71368450–71368551
DNA to multiple species	Which organism does the DNA sequence come from: AGGGGC AGCAAA CACCGG GACACA CCCATT CGTGCA CTAATC AGAAAC TTTTTT TTCTCA AATAAT TCAAAC AATCAA AATTGG TTTTTT CGAGCA AGGTGG GAAATT TTTCGAT	worm

**Table 6:** Sample question-answer pairs of the GeneHop tasks (introduced in this work).

GeneHop task	Question	Answer
SNP gene function	What is the function of the gene associated with SNP rs1318850293? Let’s decompose the question to subquestions and solve them step by step.	Predicted to enable guanylnucleotide exchange factor activity. Predicted to be involved in Rho protein signal transduction.
Disease gene location	List chromosome locations of the genes related to Hemolytic anemia due to phosphofructokinase deficiency. Let’s decompose the question to sub-questions and solve them step by step.	21q22.3
Sequence gene alias	What are the aliases of the gene that contains this sequence: GTAGAT GGAACT GGTAGT CAGCTG GAGAGC AGCATG GAGGCG TCCTGG GGGAGC TTCAAC GCTGAG CGGGGC TGGTAT GTCTCT GTCCAG CAGCCT GAAGAA GCGGAG GCCGA. Let’s decompose the question to sub-questions and solve them step by step.	SLC38A6, NAT-1, SNAT6

### Sequence gene alias:

The question template for this task is *“What are the aliases of the gene that contains this sequence:* {sequence}. *Let’s decompose the question to sub-questions and solve them step by step.”*. We find the information pages of the 50 genes used in the gene alias task in the original GeneTuring benchmark. We manually crop part of the sequence with a similar length to the sequences in the GeneTuring alignment tasks to serve as the {sequence}, and use the union of its official name and the alias set as the ground-truth answer.

## D GeneHop evaluation

### SNP gene function:

We manually evaluate all answers predicted by GeneGPT and the New Bing against the ground-truth gene functions. New Bing answers with *“I’m sorry, but I couldn’t find any information about the gene associated with SNP {snp}]. Would you like to know more about SNPs in general?”* for all questions, which we simply 0. To evaluate GeneGPT’s results, we score 1 if (a) the predicted answer exactly matches the ground-truth or (b) the gene is a non-coding, and GeneGPT’s answer mentions it; we score 0.5 if there is a partial match, and 0 otherwise.

### Disease gene location:

Following the evaluation of the gene disease association task in GeneTuring, we measure the recall of ground-truth chromosome locations. We manually evaluate all answers given by the New Bing, and consider partial match as correct. For example, if the new Bing answers “17q21” and the ground-truth answer is “17q21.2”, we still consider the prediction by new Bing correct. We automatically evaluate GeneGPT’s prediction under a more strict setting where we only consider exact matches of GeneGPT as correct.

### Sequence gene alias:

We manually evaluate all answers predicted by GeneGPT and the New Bing, and measure the recall of the ground-truth gene aliases. We only consider exact matches between the predicted alias and a ground truth alias, but ignore the case difference (e.g., ‘Myc’ and ‘MYC’ are still considered as a match).

## E Error types

### Error type 1 (E1):

Errors caused by using the wrong API or not using APIs. This only happens in disease-related tasks where the model uses the gene instead of the omin database. One example is shown in Figure 11.

### Error type 2 (E2):

Errors caused by using the right API but wrong arguments, e.g., passing terms to id. One example is shown in Figure 12.

### Error type 3 (E3):

Errors caused by not extracting the answer in the API result, most commonly seen in gene function extraction. One example is shown in Figure 13.

### Error type 4 (E4):

The model makes the right API call, but the results do not contain the answer. These questions are not answerable with the Web APIs. One example is shown in Figure 13.

### Other errors (O):

Errors that cannot be classified into E1–4 are included in this category, which are most commonly seen in BLAST-related tasks where the chromosomes are right but the specific ranges are not matched (e.g., chr8:7081648–7081782 v.s. chr8:1207812–1207946) because the original GeneTuring benchmark does not specify a reference genome.
